# Does mycorrhizal status alter herbivore-induced changes in whole-plant resource partitioning?

**DOI:** 10.1093/aobpla/plx071

**Published:** 2017-12-15

**Authors:** Colin M Orians, Sara Gomez, Timothy Korpita

**Affiliations:** Department of Biology, Tufts University, Medford, MA, USA

**Keywords:** Induction, *Manduca*, *sexta*, protein, *Solanum*, *lycopersicon*, soluble sugars, starch, total nitrogen

## Abstract

Both mycorrhizae and herbivore damage cause rapid changes in source–sink dynamics within a plant. Mycorrhizae create long-term sinks for carbon within the roots while damage by leaf-chewing herbivores causes temporary whole-plant shifts in carbon and nitrogen allocation. Thus, induced responses to herbivory might depend on the presence or absence of mycorrhizae. We examined the effects of mycorrhizal presence on induced resource partitioning in tomato (*Solanum lycopersicon*) in response to cues from a specialist herbivore *Manduca sexta*. Differences in plant size, growth and in the concentrations of carbon-based (soluble sugars and starch) and nitrogen-based (protein and total nitrogen) resources in three tissue types (apex, stem and roots) were quantified. Both mycorrhizae and simulated herbivory altered the concentrations of carbon- and nitrogen-based resources. Mycorrhizae promoted plant growth, altered sugar and starch levels. Simulated herbivory resulted in lower concentrations of most resources (sugar, starch and protein) in the rapidly growing apex tissue, while causing an increase in stem protein. There was only one interactive effect; the effects of simulated herbivory were much stronger on the sugar concentration in the apex of non-mycorrhizal plants. This clearly demonstrates that both mycorrhizal colonization and herbivore cues cause shifts in carbon- and nitrogen-based resources and further shows there is little interference by mycorrhizae on the direction and magnitude of plant responses to herbivory. Overall, our results suggest that herbivore cues, regardless of mycorrhizal status, reduce allocation to the growing apex while inducing protein storage in the stem, a possible mechanism that could increase the tolerance of plants to damage.

## Teaser

Plants simultaneously interact with beneficial and antagonistic organisms such as mycorrhizal fungi and herbivores, respectively. We studied the net effects of such interaction on plant internal resources and growth and found that while both organisms had an impact on the plant individually, the magnitude or direction of the individual effects generally did not differ when both organisms were present simultaneously. These results suggest that despite the plant carbon diverted to feed the beneficial fungi, tomato plants are able to modulate internal resource allocation that may increase the tolerance of plants to herbivore attack.

## Introduction

Throughout their development plants interact with diverse above- and below-ground organisms. The interactions range from permanent to highly ephemeral. Mycorrhizal fungi, for example, form long-term associations with their host plant, which results in shifts in nutrient acquisition, source–sink dynamics and ultimately plant growth and defence (Smith and Read 2010; Pedone-Bonfim *et al.* 2013; Gilbert and Johnson 2015; Schweiger and Müller 2015). In contrast, mobile leaf-chewing insect herbivores interact with the host plant for only a short period of time and cause transient changes in metabolism that may enhance the defence and tolerance of the host (Karban and Baldwin 1997; Orians *et al.* 2011; Schultz *et al.* 2013; Robert *et al.* 2014; Acevedo *et al.* 2015; Zhou *et al.* 2015; Johnson *et al.* 2016). In response to herbivory, for example, recently captured carbon or nitrogen can be used by the plant to produce chemical defences (Schultz *et al.* 2013) or can be moved to tissues away from the site of feeding (Babst *et al.* 2005, 2008; Gómez *et al.* 2010; Robert *et al.* 2014), and might ultimately enhance plant tolerance (Orians *et al.* 2011). Given that mycorrhizal fungi create long-term sinks for carbohydrates and that herbivory generates transient changes in the source–sink dynamics, it is important to determine if mycorrhizal status alters herbivore-induced changes in the concentration of resources within a plant, especially considering the near-ubiquity of mycorrhizal associations in natural systems.

Both ectomycorrhizal and arbuscular mycorrhizal fungi (AMF) provide mineral nutrients to the host plant and also alter the concentrations of both primary and secondary metabolites within the host (Simon *et al.* 1993; Wright *et al.* 1998; Pedone-Bonfim *et al.* 2013; Gilbert and Johnson 2015; Schweiger and Müller 2015). Pedone-Bonfim *et al.* (2013) demonstrated that the concentrations of sugars and protein, as well as various phenolics were elevated in mycorrhizal plants. Yet, there is a cost of this association; the plant must provide carbohydrates to the fungi (Simon *et al.* 1993; Wright *et al.* 1998; Smith and Read 2010); estimates vary, but 2–20 % of total primary production may be used to support the fungi (Smith and Read 2010). Thus, roots, which are already a strong sink for carbon, are made an even stronger sink by the presence of mycorrhizae. Despite these costs, there are clear benefits. Mycorrhizal plants generally have improved mineral nutrition, grow faster and may be more resistant to herbivores and pathogens (Kempel *et al.* 2010; Gilbert and Johnson 2015; Kula and Hartnett 2015). The effects of mycorrhizal status on plant tolerance to herbivory are variable (Borowicz 2013). Kula *et al.* (2005) showed that mycorrhizae increased regrowth potential after a defoliation event. Yet there are counter examples (Markkola *et al.* 2004; Garrido *et al.* 2010). Markkola *et al.* (2004) showed that the increased sink strength of mycorrhizal fungi-colonized roots increased carbon limitation after defoliation.

The effects of herbivory on source–sink dynamics are also quite pronounced (Schultz *et al.* 2013). In response to herbivory or herbivore cues, or following the application of jasmonates—plant signalling molecules induced by many herbivores, there is a general increase in the transport of recently fixed carbon away from the site of damage (Babst *et al.* 2005, 2008; Schwachtje *et al.* 2006; Henkes *et al.* 2008; Gómez *et al.* 2012; Moreira *et al.* 2012; Poveda *et al.* 2012; Ferrieri *et al.* 2013; Robert *et al.* 2014). Interestingly, nitrogen has also been shown to be mobilized in response to herbivory or simulated herbivory (Newingham *et al.* 2007; Gómez *et al.* 2010), and jasmonates have been shown to increase protein concentration in stem tissue of *Populus* (Beardmore *et al.* 2000). This increased transport of resources away from the location of damage and towards storage tissues that are inaccessible to folivores (termed induced sequestration by Orians *et al.* 2011) may be an especially important response to specialist herbivores that are adapted to consume leaves despite increases in chemical defences (Bowers 1983; Steinbrenner *et al.* 2011). Such a response could lead to increased regrowth potential (Korpita *et al.* 2014; Robert *et al.* 2014) after the threat from the herbivore has passed. Korpita *et al.* (2014) showed that damage by *Manduca sexta*, a specialist on Solanaceae, depresses shoot growth in tomatoes, but causes an increase in stem diameter, branching, and greater leaf and flower production following a simulated severe defoliation event. This suggests that an herbivore attack might cause starch and protein concentrations to increase within tissues that are inaccessible to herbivores, and would be remobilized to support regrowth.

It is possible that mycorrhizal colonization might alter plant responses to herbivory beyond their known effects on plant resistance (Pozo *et al.* 2010; Barber 2013; Minton *et al.* 2016). Mycorrhizal colonization may prevent the accumulation of resources in storage tissues since mycorrhizae are major sinks for resources. Mycorrhizae could, however, increase the observed shifts in resource allocation to inaccessible tissues since both herbivores and mycorrhizal colonization can activate or prime the jasmonate pathway (Kempel *et al.* 2010; Jung *et al.* 2012; Song *et al.* 2013; Wang *et al.* 2015).

In this study, we examine the effects of AMF and cues of *M. sexta* on induced changes in carbon- and nitrogen-based resources within different tomato plant tissue (roots, stems and apical leaves). Previous work in the tomato system has shown that (i) tissue demand strongly influences patterns of resource allocation within a plant (Bledsoe and Orians 2006; Zanne *et al.* 2006), (ii) herbivore cues increase carbon and nitrogen allocation to root tissue (Gómez *et al.* 2010), (iii) herbivores, especially cues of the *M. sexta*, cause metabolites associated with transport to increase (Steinbrenner *et al.* 2011; Gómez *et al.* 2012) and (iv) plants exposed to herbivore cues show growth suppression but increased regrowth capacity following defoliation (Korpita *et al.* 2014). Thus, this is an excellent system to test the hypothesis that the presence of AMF might alter the shifts in concentrations of resources within the plant in response to *M. sexta* damage cues. Specifically, we examined changes in the actively growing apex, in the stem (a storage tissue), and in roots, the site of AMF colonization.

## Methods

### Plant material and mycorrizhal inoculation

Tomato (*Solanum lycopersicum*, First Lady Cultivar) plants were grown in a sterile, nutrient-free 50:50 sand-zeolite medium in 10 cm pots. The plants were grown in a greenhouse under natural light supplemented with metal-halide lamps (16:8 h photoperiod), and fertilized three times a week with 50 mL of a modified Hoagland solution (Hoagland and Arnon 1938). The modified solution consisted of twice the concentration of KHPO_4_ (380 μM) to avoid the inherent phosphorus deficiency of the sand-zeolite mix.

There were two mycorrhizal treatments: inoculated (‘AMF’) and uninoculated (‘non-AMF’, *n* = 24 per group). For AMF inoculation, the same sand-zeolite medium was mixed with 50 cc of dried whole inoculum from the International Culture Collection of Vesicular Arbuscular Mycorrhizal Fungi (INVAM) (West Virginia University). The AMF species used were a 50:50 mixture of inoculum from *Glomus etunicatum* and *Glomus mosseae*. For the non-AMF treatment we used a water-only treatment. This contrasts with previous experiments that applied a filtered version (to control for the effects of bacteria) and/or an autoclaved version (to control for chemical factors) of the inoculant (Charron *et al.* 2001; Tao *et al.* 2016). As a consequence, other factors in the inoculum could have influenced our results. We note, however, that the roots were clearly colonized in the AMF treatment and that AMF plants grew faster as would be expected by the presence of AMF (see Results below). Standard methods were used to confirm colonization (Koske and Gemma 1989). Briefly, root tissue was washed and then cut into segments ~5 cm long. The root segments were cleared in a near-boiling 10 % potassium hydroxide solution for 10 min. The tissues were then washed in deionized water and placed in 2 % hydrochloric acid for 15 min. The root fragments were stained in a heated 0.05 % trypan blue stain (10 mL glacial acetic acid, 200 mL glycerol, 0.2 g trypan blue, 190 mL deionized water) for 15 min. Excess stain was rinsed off with deionized water and the roots were viewed under the microscope to confirm colonization status. Although the extent of colonization was not quantified, only AMF colonization was evident in the AMF treatment.

### Damage treatments

There were three treatments: an undamaged control and two damage treatments with eight plants per treatment. In each damage treatment, leaves 2, 3 and 4 from the top were damaged (leaf 1 was defined as the newest leaf that was over 50 % expanded). To damage the plants, a pinwheel was used to make puncture wounds along the edge of every leaflet on each leaf. After mechanical damage, a paintbrush was used to apply either 80 μL of deionized water (=‘MD’ treatment) or 80 μL *M. sexta* regurgitant/oral secretion (=‘MD+OS’ treatment) to the punctured leaflets (see Korpita *et al.* 2014 for details). This treatment was used to ensure that the shifts observed could be attributed to responses to herbivore attack and not to the loss of photosynthetic area. Caterpillar oral secretion was collected from fifth instar *M. sexta* larvae fed on tomato plants for at least 48 h and stored at −80 °C. The treatments were initiated after 8 weeks of plant growth (with and without AMF inoculation). To simulate continuous damage, these treatments were repeated for 5 days. Plants were then harvested on Day 6.

### Plant growth

At the start of the damage treatments, and again prior to harvest on Day 6, plant height and number of leaves were recorded. At harvest on Day 6, three tissues were harvested from each plant (roots, stems and the apex). The apex included the expanding shoot and all leaves that were <50 % expanded at the start of the damage treatment, and the stem tissue included all stem below the apex. The roots were washed and then blotted dry. The tissues were flash-frozen in liquid nitrogen, lyophilized, weighed and then pulverized into a fine powder using a ball mill (Kleco, Visalia, CA, USA). The tissues were stored at −80 °C until analysis.

### Chemical analyses

The concentrations of soluble sugars, starch, total nitrogen and protein were analysed in the three tissue types described above. For soluble sugars and starch, 10 mg of ground tissue was extracted in 80 % aqueous ethanol under sonication for 15 min. The resulting supernatant was removed and this process was repeated three times. The phenol sulfuric acid method described in Dubois *et al.* (1956) was used to determine soluble sugar content present in the supernatant as glucose equivalents by measuring absorbance at 487 nm in a 96-well microtiter plate reader (Bio-Rad Laboratories, Hercules, CA, USA). To determine starch concentration, the remaining pellet was digested enzymatically using aqueous 0.5 % amyloglucosidase (Sigma-Aldrich-10115), and incubated overnight at 55 °C (Haissig and Dickson 1979). This enzyme degrades starch into glucose and the resulting free sugars in the supernatant were again analysed with the phenol sulfuric acid method.

We quantified, using 5 mg of ground leaf tissue, total nitrogen using a CHN analyzer (Vario Microcube, Elementar Americas, Mt. Laurel, NJ, USA). Protein concentration was quantified using a colorimetric method (Bradford 1976; Jones *et al.* 1989). Briefly, 3 mg ground tissue was extracted in 1.5 mL of a boiling NaOH 0.1 N solution for 50 min. The concentration of protein in the supernatant was determined using a dye-binding reagent (#500-0006; Bio-Rad) followed by absorbance at 595 nm on a plate reader (Bio-Rad Laboratories, Hercules, CA, USA).

### Statistical analysis

All statistical analyses were performed using JMP version 12. A Shapiro–Wilk goodness-of-fit test was used to ensure normality. When not normally distributed, natural log transformations were done (stem starch, nitrogen and protein, and root starch).

A one-way ANOVA, with AMF status as a fixed effect, was performed to determine if AMF plants differed in height prior to the initiation of the damage treatments. At harvest, after 5 days of the damage treatments, a two-way ANOVA was used to determine the effects of AMF status and damage treatment on final plant mass and on relative height growth rate during the 5 days of damage treatment [(Day 6 height − Day 1 height)/Day 1 height].

Carbon- and nitrogen-based resources in apex, stem and root tissues were analysed separately. Because plants differed in size at the initiation of damage treatments (see Results below) for each trait, we used plant mass as a covariate when significant, resulting in either a two-way MANCOVA (for stem starch and nitrogen only; with AMF inoculation and damage treatment as fixed factors, total plant mass as the covariate) or a two-way MANOVA (for all other tissues). If the interaction effect was not significant, we reanalyzed and focused on the two main effects. *Post hoc* Tukey–Kramer tests were performed to determine differences among the three damage treatments when significant.

## Results

### Plant size

**Figure 1.**
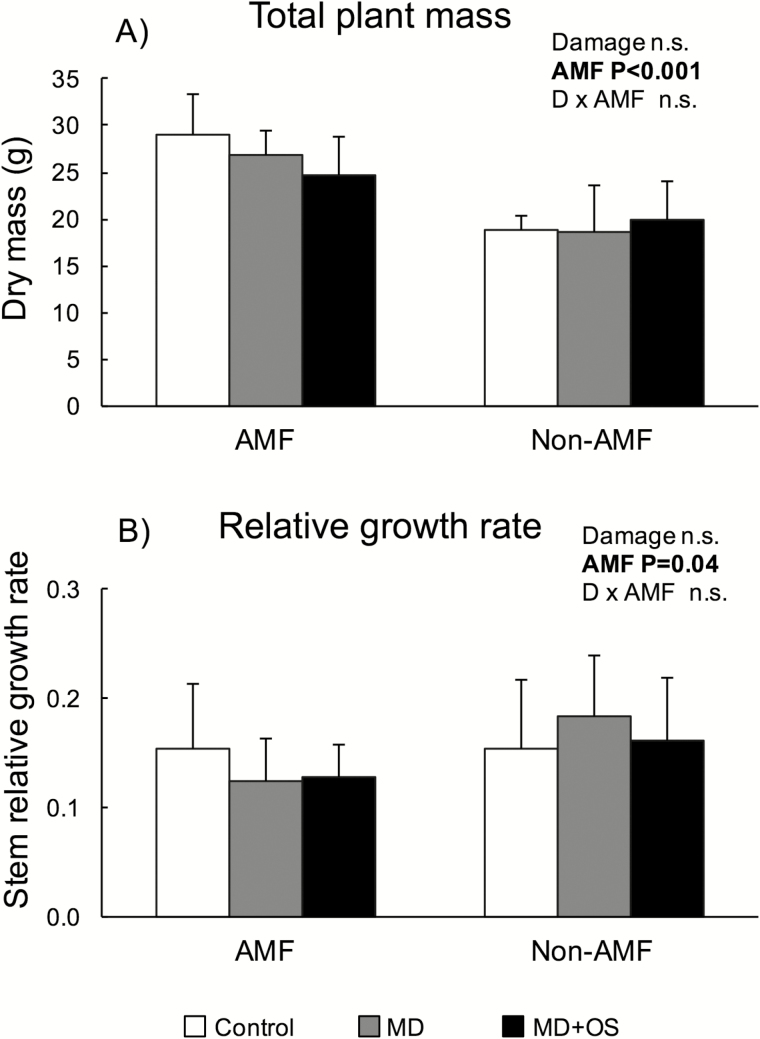
Effects of mycorrhizal status (AMF and non-AMF) and three damage treatments (control, MD, MD+OS) on (A) final plant mass at harvest, and (B) relative stem growth rate during the 5 days of damage treatments [(Day 5 − Day 1)/Day 1]. All values are means ± 1 SD.

At harvest, AMF plants weighed 40 % more than non-AMF plants (*F*_1, 46_ = 48.7, *P* < 0.001; Fig. 1A). The presence of AMF also resulted in plants that were 30 % taller at the start of the damage treatments (*F*_1, 46_ = 40.1, *P* < 0.001). There was no effect of damage treatment (*F*_2, 41_ = 0.79, *P* = 0.46) or its interaction with AMF status (*F*_2, 41_ = 2.09, *P* = 0.14) on final plant mass.

For stem growth rate during the 5 days of damage treatment (Fig. 1B), there was an effect of AMF status (*F*_1, 42_ = 4.41, *P* = 0.04) but no effect of damage treatment (*F*_2, 41_ = 0.17, *P* = 0.84) or their interaction (*F*_2, 41_ = 1.32, *P* = 0.28). Non-AMF plants exhibited 20 % higher relative growth rate than AMF plants.

### Sugars

**Figure 2.**
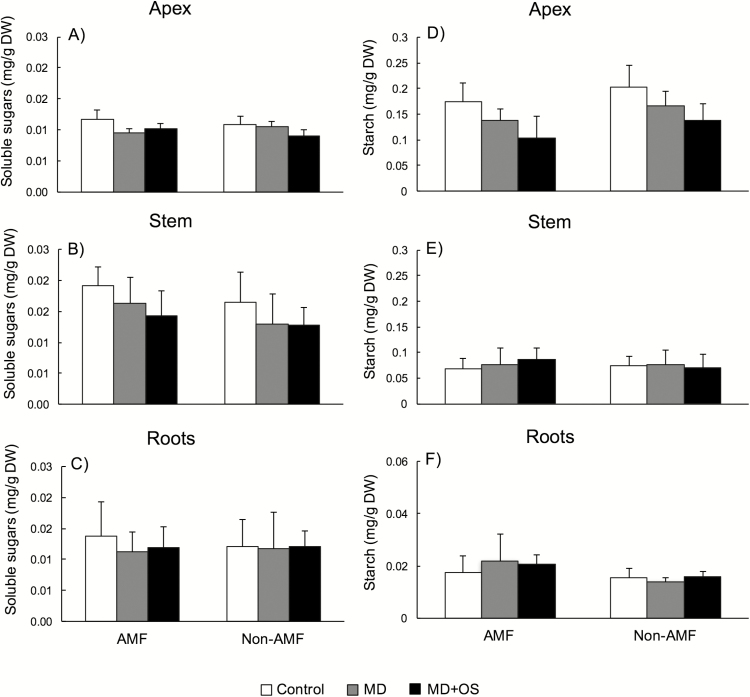
Effects of mycorrhizal status and damage treatment on the concentrations (mg g^−1^ dry weight) of carbon-based resources, total sugar concentration in apex, stem and root tissue (A–C), and starch concentration in apex, stem and root tissue (D–F). All values are means ± 1 SD.

Sugar concentrations varied across tissue types (Fig. 2A–C). Overall, soluble sugars were lowest in apex tissue and highest in stem tissue. Both AMF status and damage treatments affected sugar concentrations but the effects varied by tissue type (Table 1).

**Table 1.** Effects of AMF colonization (inoculated and uninoculated), damage (control, mechanical damage, and mechanical damage + oral secretions) and their interaction on the concentrations of sugars, starch, total N and protein in apex, stem and root tissues in tomato. Within each trait a two-way MANOVA (with AMF inoculation and damage treatment as fixed factors) was used unless total plant mass (see *) was found to be correlated with those traits (in this case a MANCOVA was employed). Significant results (*P* < 0.05) are highlighted in bold.

Trait	Tissue	Source of variation					
		AMF		Damage		AMF × damage	
		*F*_1, 42_	*P*	*F*_2, 42_	*P*	*F*_2, 42_	*P*
Sugar	Apex	1.50	0.23	9.77	**<0.001**	4.92	**0.01**
	Stem	4.42	**0.04**	4.91	**0.01**	0.21	0.81
	Root	0.06	0.80	0.41	0.66	0.26	0.77
Starch	Apex	8.78	**0.005**	14.62	**<0.001**	0.06	0.94
	Stem	2.91	0.09	0.79*	0.46	1.55	0.22
	Root	9.82	**0.003**	0.52	0.59	1.14	0.33
Total N	Apex	47.36	**0.001**	2.75	0.08	2.83	0.07
	Stem	23.08	**<0.001**	0.84*	0.43	0.80	0.45
	Root	11.15	**0.001**	0.33	0.72	1.89	0.16
Protein	Apex	0.05	0.83	5.48	**<0.01**	0.26	0.77
	Stem	0.10	0.75	5.85	**0.006**	0.57	0.56
	Root	1.54	0.22	1.20	0.31	0.02	0.98

In the apex tissue, there was no effect of AMF but a significant effect of damage and AMF × damage treatment interaction (Table 1, Fig. 2A). In AMF plants, both damage treatments had lower sugars (23 and 14 %, respectively) than controls (*P* = 0.03), but in non-AMF plants, MD+OS plants had 18 % lower (*P* < 0.04) sugar concentration than both control and MD plants, which did not differ from one another (*P* = 0.85). For stem tissue, only the main effects were significant (Table 1, Fig. 2B). Arbuscular mycorrhizal fungi plants had 13 % higher soluble sugars (*F*_1, 42_ = 4.42, *P* = 0.04). In contrast, damage treatment generally reduced soluble sugars. Control plants had 34 % higher concentrations than MD+OS plants (*P* = 0.01). Plants in the MD treatment were intermediate and did not differ from control (*P* = 0.07) or MD+OS (*P* = 0.72). For root tissue, the main effects and their interaction were not significant (Table 1, Fig. 2C).

### Starch

Starch concentrations also varied across tissue types (Fig. 2D–F). Overall, starch concentrations were highest in the apex and stem and lowest in the root. Both AMF status and damage treatments affected starch concentration but the effects varied by tissue type (Table 1).

In apex tissue (Table 1, Fig. 2D), the main effects of AMF and damage were significant but their interaction was not. Overall, AMF plants had 21 % less starch. In contrast, damage treatments reduced apex starch. Control plants had 23 and 56 % higher starch than MD (*P* < 0.001) and MD+OS plants (*P* = 0.02), respectively, and MD+OS plants were significantly lower than MD plants (*P* = 0.03). For stem tissue (Table 1, Fig. 2E), there was no effect of AMF status, damage treatment or their interaction. Although not significant, AMF plants tended (*P* = 0.09) to have less starch in their stems. For root tissue (Table 1, Fig. 2F), there was an effect of AMF status, AMF plants had 32 % higher starch compared to non-AMF plants, but no effect of damage or their interaction.

### Total nitrogen

**Figure 3.**
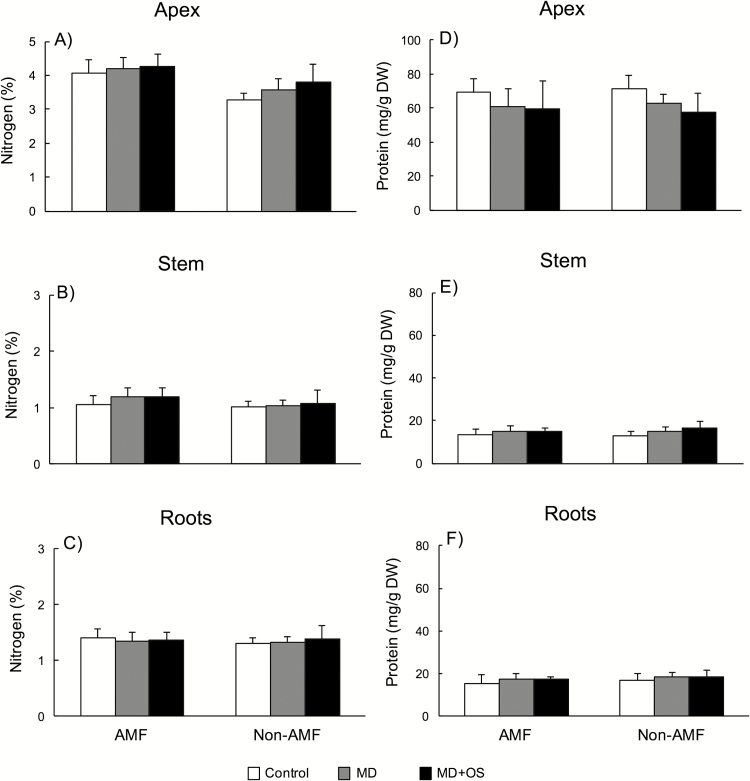
Effects of mycorrhizal status and damage treatment on the concentrations (mg g^−1^ dry weight) of nitrogen-based resources, total nitrogen concentration in apex, stem and root tissue (A–C), and protein concentration in apex, stem and root tissue (D–F). All values are means ± 1 SD.

Nitrogen concentrations varied across tissue types and depended on total plant mass (Fig. 3A–C). Overall, nitrogen concentrations were highest in apex tissue and lowest in the stem tissue. Both AMF status and damage treatments affected nitrogen levels but the effects varied by tissue type (Table 1).

In apex tissue, only AMF status affected total nitrogen concentrations (Table 1, Fig. 3A). Arbuscular mycorrhizal fungi plants had 21 % higher nitrogen than non-AMF plants. For stem tissue, again only AMF status was significant (Table 1, Fig. 3B). Arbuscular mycorrhizal fungi plants had 9 % higher N than non-AMF plants. For root tissue (Table 1, Fig. 3C), once again only AMF status affected total nitrogen. Roots of AMF plants had 20 % higher N than those of non-AMF plants when controlling for total plant mass.

### Protein

Protein concentrations also varied across tissue types (Fig. 3D–F). Overall, protein was much higher in apex tissue and similar in root and stem tissue. Arbuscular mycorrhizal fungi status did not affect protein levels but the damage treatments did in a tissue-specific manner (Table 1).

In apex tissue, only damage treatment affected protein concentrations (Table 1, Fig. 3D). Control plants had 20 % higher protein concentration than MD+OS plants (*P* = 0.006). MD plants were intermediate and not significantly different from control (*P* = 0.07) or MD+OS (*P* = 0.58). For stem tissue, once again only damage treatment affected protein concentrations (Table 1, Fig. 3E). In contrast to the apex, stem protein was higher in the damage treatments compared to the controls. MD and MD+OS plants had 15 and 19 %, respectively, higher protein than the control plants (MD vs. control, *P* = 0.03; MD+OS vs. control, *P* < 0.01), but did not differ (*P* = 0.88). For root tissue, there was no effect of mycorrhizal status, damage treatment or their interaction on protein concentration (Table 1, Fig. 3F).

## Discussion

Both mycorrhizae and herbivores are known to alter within-plant allocation of resources but few studies have examined the effects of the two together. Overall, the effects of AMF inoculation (increased growth pre-damage, changes in carbon-based resources and higher overall nutrient status) were as expected. While these changes were likely due to the effects of AMF, we acknowledge that other factors in the inoculum such as bacteria could have influenced our results. In contrast, the effects of simulated herbivory were quite novel. Simulated herbivory lowered the concentrations of resources, especially sugar, starch and protein, in the rapidly growing apex, while the concentration of protein in the stem was ~15 % higher compared to undamaged plants. These results are consistent with the hypothesis that herbivores can induce storage in tissues unavailable to the foraging insects (Orians *et al.* 2011) and this change might explain the higher stem diameter and higher regrowth potential of previously damaged plants (Korpita *et al.* 2014).

The interactive effects of AMF and damage were limited. We had hypothesized that mycorrhizae, as strong sinks for carbohydrates (Smith and Read 2010) and manipulators of phytohormone signalling (Pozo and Azcón-Aguilar 2007), might alter the patterns of herbivore cue-induced changes in plant growth and chemistry (Gómez *et al.* 2010, 2012; Steinbrenner *et al.* 2011). We found evidence of a trade-off in growth as the relative stem growth rate of AMF plants during a damage treatment was lower than non-AMF plants. Despite this apparent trade-off, we found little interference by mycorrhizae on the direction and magnitude of chemical responses to herbivore cues. Arbuscular mycorrhizal fungi status only altered the induced changes in soluble sugars in the actively growing meristem (apex).

### Effects of AMF

Arbuscular mycorrhizal fungi plants were 30 % taller, and had ~15 % higher nitrogen levels in above-ground tissues. Arbuscular mycorrhizal fungi colonization caused plant-wide shifts in carbon-based resources, and to a lesser extent, nitrogen-based resources. These results are consistent with previous research showing, despite variation among species, mycorrhizae induce shifts in the growth and metabolome of their host plant (Klironomos 2003; Schweiger *et al.* 2014; Schweiger and Müller 2015; Tao *et al.* 2016).

Because mycorrhizae are strong sinks for carbohydrates (Smith and Read 2010), whole-plant changes in sugars and starches were expected. Our results suggest that the fungi increased carbohydrate movement from apical tissues towards roots but once at the roots it was incorporated into starch or transported to the fungal hyphae as shown by lower foliar starch levels and high soluble sugars in the apex and higher starch concentration in the roots. Soluble sugars were also significantly higher in stems of AMF plants. Although AMF colonization did not alter levels of protein, it did alter the concentration of nitrogen in the different tissues. Total nitrogen was higher in the apex and stem, indicating a general increase in plant nutrient status. How the extent of colonization might alter these patterns was not explored but deserves further study.

### Effects of damage

As hypothesized, we found strong evidence that damage, especially when associated with *M. sexta* oral secretion, causes shifts in resource allocation. Compared to control plants, simulated herbivore damage, especially damage plus oral secretions, had lower concentrations of sugars, starch and protein in the apex, while protein concentrations were higher in the stem. This result builds on previous findings demonstrating that herbivore cues can inhibit new growth (Korpita *et al.* 2014), and can shift resource transport towards stem and roots (Babst *et al.* 2005, 2008; Schwachtje *et al.* 2006; Gómez *et al.* 2010; reviewed by Zhou *et al.* 2015). Total nitrogen was only higher in the apex of damaged plants. Perhaps this nitrogen was used to produce nitrogen-based defences, enzymes or secondary metabolites like tomatine, to protect valuable young leaves.

We suggest the induction of protein in stem tissue could be a mechanism to store valuable nitrogen in tissues inaccessible to foraging herbivores. Total nitrogen levels showed a similar but non-significant trend. Vegetative storage proteins are important to nitrogen accumulation and mobilization (Lee *et al.* 2014) and one study showed that jasmonates induce bark storage protein in *Populus* (Beardmore *et al.* 2000). Thus, local herbivore-induced increases in transport amino acids (Steinbrenner *et al.* 2011; Gómez *et al.* 2012) could then be moved to the stem and be incorporated into transient vegetative storage protein. A second, non-mutually exclusive, explanation is that simulated herbivory induces an increase in photosynthetic protein, RuBisCO, within stems of tomato. Since the protein assay used here is non-specific, proteomic studies are needed to determine the identity of the proteins induced. Interestingly, there was no effect of our treatments on starch levels, indicating that changes in nitrogen metabolism may be much more important.

Induced increases in stem protein might be particularly beneficial against specialist herbivores that generally perform better on mycorrhizal plants (Koricheva *et al.* 2009), and are well adapted to the chemical defences of their host plant (Bowers 1983). In our previous work, Steinbrenner *et al.* (2011) showed that tomato responses to the generalist, *Helicoverpa zea*, were quite distinct from that of the specialist, *M. sexta*. *Helicoverpa Zea*-induced changes were more consistent with defence induction while *M. sexta*-induced changes were more related to the induction of tolerance. Interestingly, resources in the roots were largely unchanged in response to damage. Thus, we might not expect an increase in stem protein in response to a more generalist herbivore.

Lastly, the lack of changes within roots is noteworthy. In wild tobacco, Schmidt *et al.* (2015) found no change in carbon transport to roots in response to herbivory. Perhaps the stem is a more important site of resource accumulation, especially since plants are also threatened by root herbivores (Robert *et al.* 2014).

### Interactions between AMF and damage

We had predicted that the presence of AMF would shift the way resources were allocated in response to simulated herbivory, either through an increase in sink strength or cross-talk between the pathways responsible for the shifts. While AMF status did alter damage-induced soluble sugar levels in the apex, it did not affect any of the measured resources in stem tissue.

Sugar concentrations in MD+OS-treated apex tissues, in the absence of AMF, were much lower than control apices. Since jasmonates and other herbivore cues induce carbon export (Gómez *et al.* 2010, 2012, Machado *et al.* 2013, 2016), this may indicate that AMF might interfere with damage-induced export of recently fixed carbon. Given that different sugars might mediate plant–herbivore interactions, future studies characterizing the response of different sugars and of resistance are needed.

Importantly, herbivore cue-induced changes in starch, protein and nitrogen were independent of AMF status. Tao *et al.* (2016) suggests that the effect of AMF on plant responses to herbivory is correlated with their effect on nutrient status, biomass allocation and growth rate in the plant. The lack of an AMF by damage interaction in our study suggests that damage-induced protein sequestration will not depend upon AMF status.

## Conclusions

These results indicate that both AMF and non-AMF tomato plants respond to herbivore cues by accumulating resources, especially protein, in tissues inaccessible to herbivores. This shift could provide a mechanism behind the greater regrowth capacity of previously damaged plants (Korpita *et al.* 2014). Examining the effects of herbivores and their cues on protein dynamics and tolerance deserves further study. Our results also suggest that soluble sugars vary as a function of both AMF status and damage. An exploration of trade-offs between damage and mycorrhizal status on foliar sugars would benefit from a more detailed examination of individual soluble sugars and resistance.

## Sources of Funding

The project was supported by the National Research Initiative (or the Agriculture and Food Research Initiative) of the USDA National Institute of Food and Agriculture, Grant Number # 2007-35302-18351 to C.M.O. Any opinions, findings, and conclusions or recommendations expressed in this material are those of the authors and do not necessarily reflect the views of the United States Department of Agriculture.

## Contributions by the Authors

All authors contributed to the design of the experiment. T.K. performed the research as part of his senior thesis; all authors contributed to data analysis and interpretation; and C.M.O. wrote the manuscript with significant input of all authors.

## Conflict of Interest

None declared.
